# COHORT DIFFERENCES IN SOCIAL TIES TO FAMILY AND FRIENDS

**DOI:** 10.1093/geroni/igac059.545

**Published:** 2022-12-20

**Authors:** Won Choi, Linda Waite

**Affiliations:** University of Chicago, Chicago, Illinois, United States; National Opinion Research Center (NORC at Chicago), Chicago, Illinois, United States

## Abstract

Dramatic changes in family life may have altered the structure and quality of social ties to family and friends. However, little is known about whether and how social relationships vary between older adults from different cohorts. Using data from the National Social Life, Health, and Aging Project, we compared social network composition and social support between older adults at ages 57 to 67 from the Silent Generation cohort (N=2,316) and the Baby Boom cohort (N=1,500). Compared with the Silent Generation cohort, the Baby Boom cohort had significantly higher odds of not listing any kin in their core discussion network. There were no cohort differences in proportion of friends in the network. The Baby Boom cohort also reported lower levels of family and friend support than their counterparts. Results suggest that the Baby Boom cohort is more socially disconnected from friends and particularly family compared with the Silent Generation cohort.

